# ISL1 Promotes Pancreatic Islet Cell Proliferation

**DOI:** 10.1371/journal.pone.0022387

**Published:** 2011-08-04

**Authors:** Ting Guo, Weiping Wang, Hui Zhang, Yinan Liu, Ping Chen, Kangtao Ma, Chunyan Zhou

**Affiliations:** Department of Biochemistry and Molecular Biology, School of Basic Medical Sciences, Key Laboratory of Molecular Cardiovascular Sciences, Ministry of Education of China, Peking University, Beijing, China; Vanderbilt University Medical Center, United States of America

## Abstract

**Background:**

Islet 1 (ISL1), a LIM-homeodomain transcription factor is essential for promoting pancreatic islets proliferation and maintaining endocrine cells survival in embryonic and postnatal pancreatic islets. However, how ISL1 exerts the role in adult islets is, to date, not clear.

**Methodology/Principal Findings:**

Our results show that ISL1 expression was up-regulated at the mRNA level both in cultured pancreatic cells undergoing glucose oxidase stimulation as well in type 1 and type 2 diabetes mouse models. The knockdown of ISL1 expression increased the apoptosis level of HIT-T15 pancreatic islet cells. Using HIT-T15 and primary adult islet cells as cell models, we show that ISL1 promoted adult pancreatic islet cell proliferation with increased c-Myc and CyclinD1 transcription, while knockdown of ISL1 increased the proportion of cells in G_1_ phase and decreased the proportion of cells in G_2_/M and S phases. Further investigation shows that ISL1 activated both c-Myc and CyclinD1 transcription through direct binding on their promoters.

**Conclusions/Significance:**

ISL1 promoted adult pancreatic islet cell proliferation and probably by activating c-Myc and CyclinD1 transcription through direct binding on their promoters. Our findings extend the knowledge about the crucial role of ISL1 in maintaining mature islet cells homeostasis. Our results also provide insights into the new regulation relationships between ISL1 and other growth factors.

## Introduction

Pancreatic islets in mouse embryos are developed between embryonic day (E) 13.5 and 15.5 [1], [2]. After birth, the number of islet cells is determined by the balance of cell renewal and cell loss [3]. The dynamic change of islet cells number is essential in maintaining euglycemia and thus it is important to understand how islet cells balance in the adult pancreas is achieved, specifically the mechanisms involved in stimulating pancreatic islet cells growth and preventing pancreatic islet cells from apoptosis. The growth rate of pancreatic islet cells is normally low but changes in response to different stimuli. The cell death or apoptosis is also an important factor in maintaining the appropriate number of islet cells. Increasing reactive oxygen species (ROS) concentration is one of the major causes to induce apoptosis of cells. It is continuously derived from glucose metabolism and cannot be effectively eliminated by endogenous antioxidant enzymes. Pancreatic islet cells undergo apoptosis in either physiological or pathological conditions. There is also an evidence that pre-existing β-cells are the major source of new β-cells during adult life span and after pancreatectomy in mice [4]. However, the exact mechanisms involved in the regulation of these processes are not yet clarified. In particular, the factors involved in these important physiological or pathological conditions are not fully identified.

Insulin gene enhancer binding protein-1 (ISL1), which belongs to LIM homeobox gene family, was first discovered and cloned in 1990 [5]. ISL1 is mainly expressed in adult islet endocrine cells (α, β, γ, ε) as well in the central nervous system [6], [7]. As a key transcription factor, the functions of ISL1 involve cell fate specification and embryonic development. In pancreas its function is twofold: 1) control the four endocrine islet cell lineages development and 2) control of dorsal pancreas mesenchyme development. Complete loss of dorsal pancreatic mesenchyme and endocrine islet cells was found in ISL1 knock-out mice embryos. It has also been shown that ISL1 could regulate the expression of several islet specific genes, such as proglucagon/glucagon (α cells), somatostatin (γ cells), amylin (δ cells) and insulin (β cells), although it is not the master regulator for these genes [8], [9], [10], [11]. However, it is not clear whether ISL1 plays more important roles rather than the regulation of these endocrine hormones secretion in postnatal pancreatic islets. Recent studies demonstrate that ISL1 is required for proliferation, migration and survival of cardiac progenitor cells [12]. It also promotes proliferation and repairing of injured motor neurons [13], [14]. Overexpressing ISL1 in endothelial cells and mesenchymal stem cells can promote blood vessel formation [15]. ISL1 is also involved in the establishment of pancreatic endocrine cells during the secondary transition (E13.5–E15.5) and controls the proliferation and survival of endocrine cells during embryonic islet developmental stage [16]. However, the roles of ISL1 in adult islets are yet not clear.

Based on reports that ISL1 can promote some types of cell proliferation as mentioned above, we designed this study in order to investigate whether ISL1 plays roles in maintaining the balance of islet cell renewal and cell loss. We show that ISL1 can promote adult pancreatic islet cells proliferation and attenuate cell apoptosis against oxidative stress. The mechanism involving ISL1 in promoting adult pancreatic islet cells proliferation includes the direct activation the cell autonomous factors c-Myc and CyclinD1. Our findings advance our understanding of the roles of ISL1 in adult pancreas and provide insights into the regulation of adult pancreatic cell proliferation.

## Results

### ISL1 was highly expressed in adult pancreatic islets

**Figure 1:**
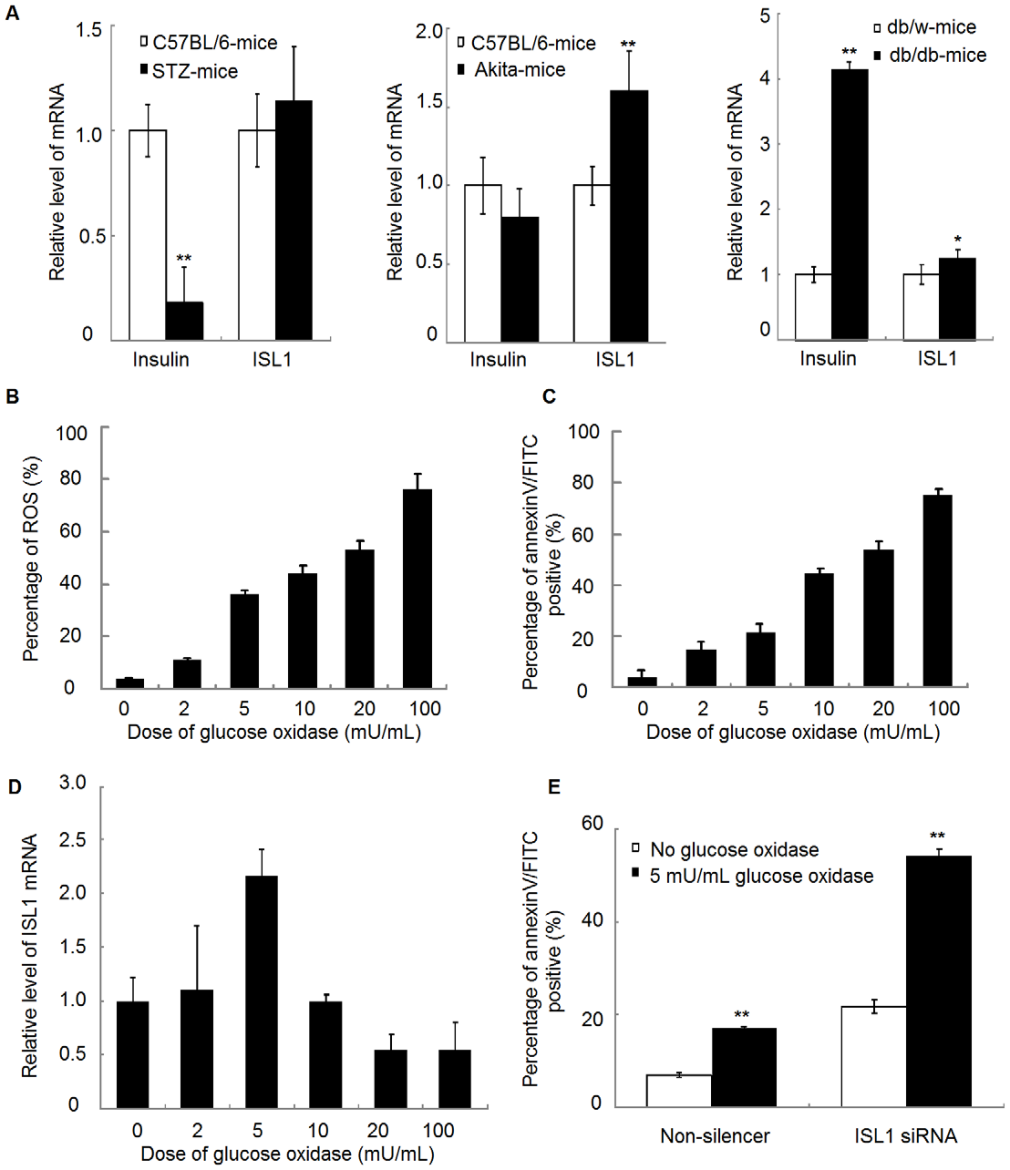
ISL1 was highly expressed in adult pancreatic islets and could reduce apoptosis in HIT-T15 cells. (A) Relative mRNA expression level of insulin in pancreatic islets from STZ mice (n = 6), Akita mice (n = 5) and db/db mice (n = 6) were measured by real-time RT-PCR. C57BL/6 mice (n = 6) were used as controls to STZ mice and Akita mice, db/w mice (n = 6) were as controls to db/db mice. Each bar represents mean ± SD (**p<0.01, *p<0.05, vs. the controls). (B) Level of ROS production was measured by flow cytometry analysis in HIT-T15 cells treated with glucose oxidase (GO) at various concentrations (0–100 mU/mL) for 4 h. (C) Level of apoptosis rate was measured by flow cytometry analysis in HIT-T15 treated with GO at various concentrations (0–100 mU/mL) for 4 h. (D) Relative level of ISL1 mRNA expression in HIT-T15 cells treated with different GO concentrations was examined by real-time RT-PCR. (E) Level of apoptosis rate was measured by flow cytometry analysis in stable ISL1 knockdown HIT-T15 cells treated with or without 5 mU/mL GO. Each bar represents mean ± SD from three samples (**p<0.01, vs. the control).

The important role of ISL1 in pancreatic islet development has been well established. However, its role in adult pancreatic islets remains unclear. The high level of ISL1 expression in adult pancreatic islets indicates that ISL1 must play important tissue specific roles. We previously reported that ISL1 enhances the transcriptional activation of the insulin gene *in vitro* [17]. In order to explore the biological functions of ISL1 in adult pancreatic islets, we detected the expression of ISL1 in different diabetes animal models. To our surprise, the increasing expression of ISL1, compared to normal control mice, was detected in all diabetes models, which is not parallel to the expression of insulin (Fig. 1A). The results indicate that ISL1 may play other roles in diabetic mice other than regulating insulin.

### ISL1 reduced apoptosis in pancreatic HIT-T15 cells

It has been reported that ISL-1 controls the proliferation and survival of endocrine cells in postnatal pancreas [16]. Considering that pancreatic islet cells are challenged by continuous oxidative stress derived from the glucose metabolism throughout life, we suspected that ISL1 plays a role in anti-oxidative stress mechanism. We used glucose oxidase (GO) to generate H_2_O_2_ from glucose, mimicking oxidative stress in HIT-T15 (a pancreatic β-cell line) cells. The flow cytometry (FCM) results showed that GO strongly promoted the production of ROS (Fig. 1B and Fig. S1) and increased the level of apoptosis (Fig. 1C and Fig. S2), in a dose-dependent manner (0–100 mU/mL). Real-time RT-PCR results showed that GO also dynamically changed the level of ISL1 expression, with a peak at 5 mU/mL of GO (Fig. 1D).

To further confirm the anti-apoptosis role of ISL1, ISL1-specific siRNA (ISL1-siRNA) was designed and transferred into HIT-T15 cells. The expression of ISL1 at both mRNA and protein levels was significantly reduced by ISL1-siRNA compared to non-silence siRNA (data not shown). The FCM result showed that knockdown of ISL1 could increase HIT-T15 cells apoptosis three folds regardless GO stimulation (Fig. 1E). These results implied that ISL1 could protect cells against apoptosis under physiological or oxidative stress conditions.

### ISL1 promoted pancreatic islet cells proliferation

**Figure 2:**
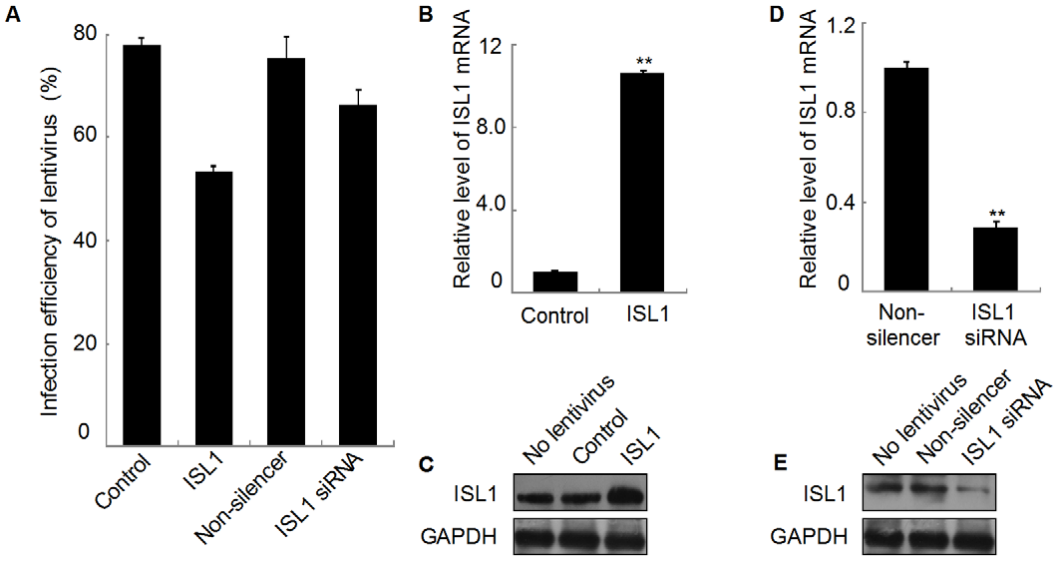
The expression of ISL1 was altered in adult islet mass by ISL1 overexpression or knockdown. (A) Infection efficiency (as indicated by the percentage of GFP positive cells in gated cells) of ISL1 overexpression lentivirus or ISL1-siRNA lentivirus were detected by flow cytometry analysis after 72 h infection. Real-time RT-PCR (B, D) and Western blotting (C, E) results showed the expression level of ISL1 in ISL1 overexpressed (B and C) islet cells and in ISL1 knockdown (D and E) islet cells. Data represent 3 independent experiments, each performed in triplicate. Each bar represents mean ± SD (**p<0.01, vs. the control). Lentivirus without any insert was used as a control.

To further define whether ISL1 plays a role in adult islets, islet mass were isolated from adult Sprague-Dawley (SD) rats and infected with ISL1 overexpressing lentivirus or ISL1-siRNA lentivirus (multiplicity of infection, MOI = 10). The infection efficiency reached approximately 53% and 66%, respectively (Fig. 2A). Real-time PCR and Western blotting results showed that the expression level of ISL1 was ameliorated ten folds in ISL1 overexpressed islet cells (Fig. 2B, 2C) and was attenuated to 30% (Fig. 2D, 2E) in ISL1 knockdown islet cells, which provided a model for further study.

**Figure 3:**
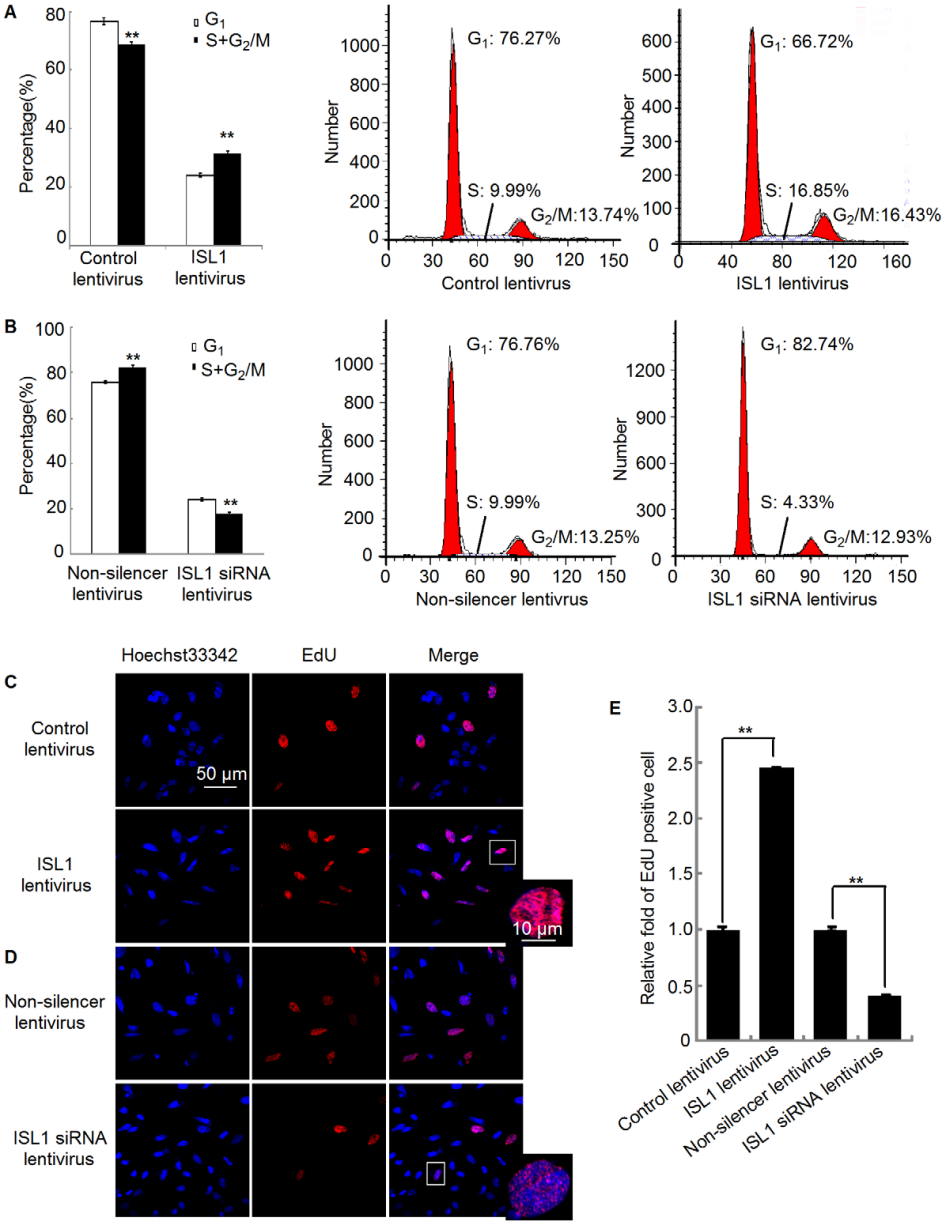
ISL1 promoted proliferation of adult pancreatic cells. Adult pancreatic islet cells infected with ISL1 lentivirus (A) or ISL1-siRNA lentivirus (B) were subjected to cell cycle analysis by flow cytometry. The data represent 3 independent experiments. The representative cytometric results from these experiments are shown. EdU incorporation assay was analyzed by confocal microscopy (scale bar, 50 µm; scale bar in magnified field, 10 µm) in adult islets infected with ISL1 lentivirus (C) or ISL1-siRNA lentivirus (D). (E) The EdU incorporation rate was expressed as the ratio of EdU positive cells to total Hoechst33342 positive cells. Each bar represents mean ± SD from 3 samples (**p<0.01, vs. the control).

To examine the impact of ISL1 on the proliferation of adult pancreatic cells, the cell cycle profile was analyzed using propidium iodide staining and flow cytometry. Compared with the control (infected with control lentivirus), ISL1 overexpression was associated with a decreased cell population in G_0_/G_1_ phases (from 76.27±1.17% to 66.72±1.62%) and an increased cell population in the G_2_/M and S phases (Fig. 3A). Conversely, adult islet mass exposed to ISL1-siRNA lentivirus exhibited an increase in the proportion of cells in G_1_ phase (from 76.76±0.67% to 82.74±0.92%) and a decrease in the proportion of cells in G_2_/M and S phases (Fig. 3B). These data indicate that ISL1 plays a role in promoting adult pancreatic cells proliferation.

Subsequently, we employed the EdU incorporation assay, a more sensitive and specific method [18], [19], to further define the function of ISL1 in promoting cell proliferation. The number of EdU positive cells was increased by 2.5 folds in ISL1 overexpressing adult islets cells relative to control cells (Fig. 3C, 3E). More importantly, the number of EdU positive cells in ISL1-siRNA lentivirus-infected cells was reduced by 40% relative to that of the cells infected with non-silencer siRNA lentivirus (Fig. 3D, 3E). These results indicate that the knockdown of ISL1 could inhibit the proliferation of adult islets *in vivo*, while the overexpression of ISL1 promotes adult pancreatic islet cells proliferation.

**Figure 4:**
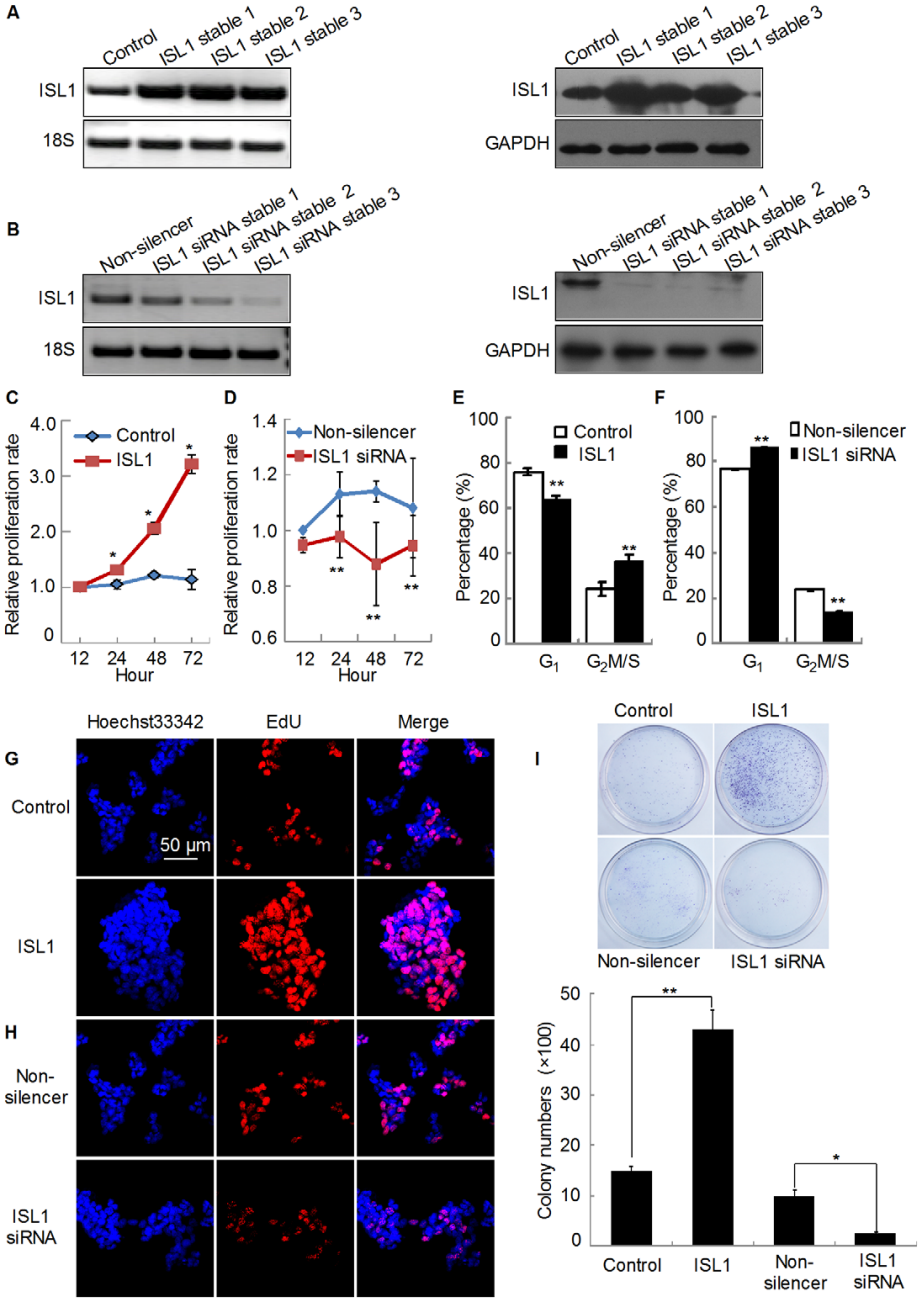
ISL1 promoted proliferation of HIT-T15 cells. The expression of ISL1 in stable overexpression (A) or knockdown (B) HIT-T15 cell lines was examined by RT-PCR and Western blotting. The cell proliferation was determined by CCK-8 analysis. A total of 1×10^3^ cells (either stably overexpressing (C) ISL1 or stably knockdown (D) ISL1) per well were seeded in 96-well plate and measured for their proliferation after 12 h, 24 h, 48 h, and 72 h. The data represent 3 independent experiments, each performed in triplicate. Each bar represents mean ± SD (**p<0.01, *p<0.05, vs. the control). The cell cycle profile was analyzed by flow cytometry in ISL1 (E) and ISL1-siRNA (F) stable HIT-T15 cell lines. EdU incorporation was detected by confocal microscopy (scale bar, 50 µm) in ISL1 (G) and ISL1-siRNA (H) stable cells. (I) Stable cells were maintained in G418 or puromycin-containing medium for 21 days before staining with crystal violet and counting for colony numbers. Each bar represents mean ± SD from 3 samples (**p<0.01, *p<0.05, vs. the control).

We also constructed a stable ISL1 overexpressing HIT-T15 cell line with the pcDNA3.1-ISL1 expression plasmid and a stable ISL1 knockdown HIT-T15 cell line with ISL1-siRNA. RT-PCR and Western blotting results showed that both overexpression (Fig. 4A) and knockdown (Fig. 4B) were established successfully in cell lines. Then, cell proliferation was determined by CCK-8 analysis. As shown in Fig. 4C, stable transfection of pcDNA3.1-ISL1 expression plasmid promoted the proliferation of HIT-T15 cells three folds relative to that of with pcDNA3.1 (control) after 72 h culture. As expected, the knockdown of ISL1 inhibited cells growth by 20% compared to inhibition with non-silencer siRNA after 48 h culture (Fig. 4D). EdU assay also showed more EdU positive cells in ISL1 overexpressing cells (Fig. 4G) and less EdU positive cells in ISL1 knockdown (Fig. 4H) cells, indicating that ISL1 promoted cell proliferation. These results are well in agreement with cell-cycle analysis that showed a decrease (from 75.94±1.45% to 63.78±1.76%) of the percentage of cells in G_0_/G_1_ phase and an increase (from 24.05±1.45% to 36.21±1.76% ) in the G_2_/M and S phases in ISL1 stable HIT-T15 cells (Fig. 4E). In contrast, ISL1 knockdown increased cell population in G_0_/G_1_ phase from 76.54±0.28% to 86.27±0.56% and decreased cell population in the G_2_/M and S phases from 23.45±0.28% to 13.73±0.56% (Fig. 4F). Colony formation assays revealed that ISL1 overexpressing cells resulted in a significant increase in colony number compared with the control cells; while ISL1 knockdown resulted in a significant decrease in colony number (Fig. 4I). These results further confirm that ISL1 promotes pancreatic islet cells proliferation.

### ISL1 stimulated islet cells proliferation through the regulation of cell cycle regulators

**Figure 5:**
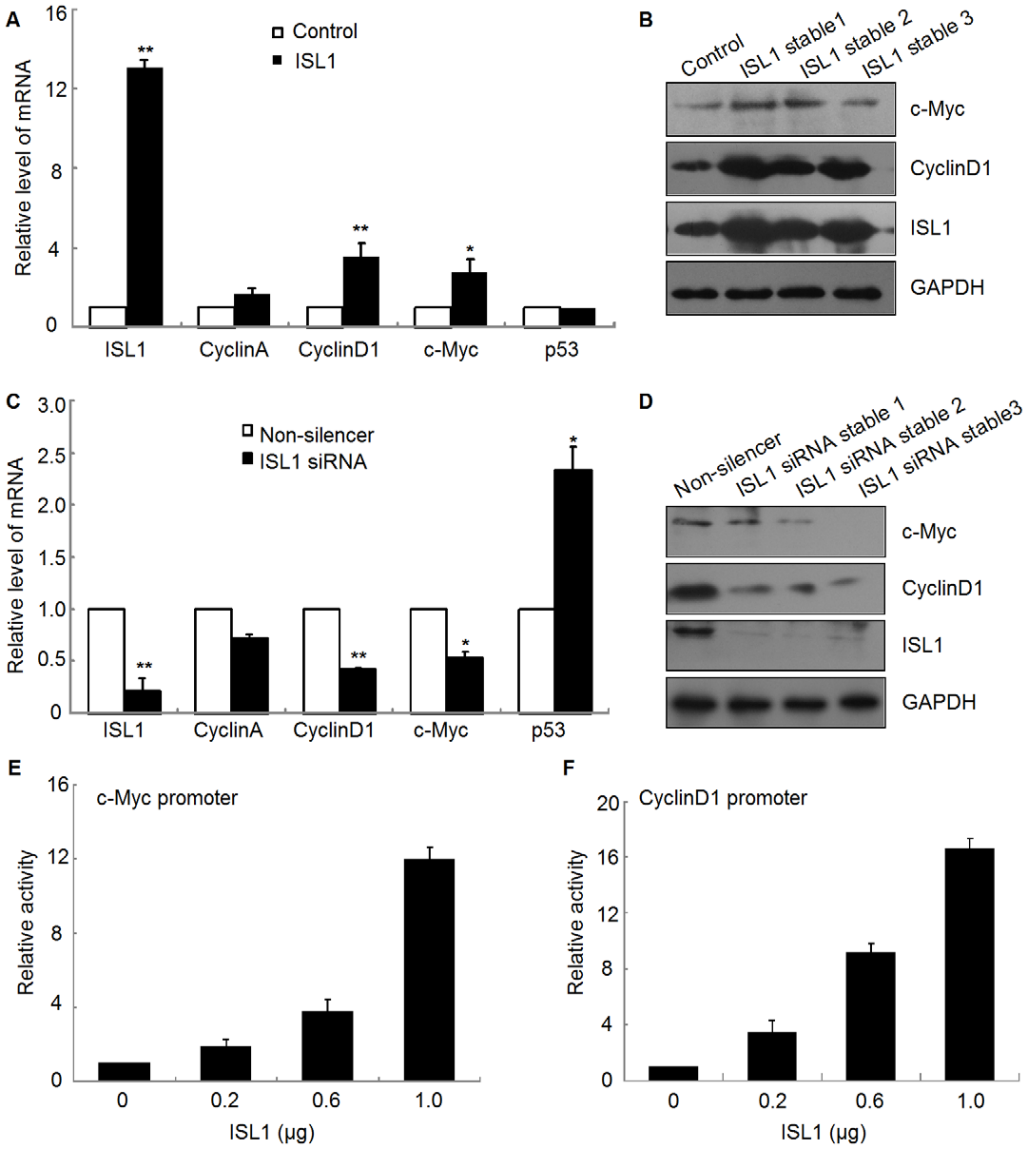
ISL1 promoted the expressions of c-Myc and CyclinD1. The mRNA level (A, C) and protein level (B, D) of c-Myc and CyclinD1, Cyclin A, p53 were analyzed by real-time RT-PCR and Western blotting in ISL1 stable overexpression HIT-T15 cells (A and B) and ISL1 knockdown HIT-T15 cells (C and D). The transcriptional activity of ISL1 was analyzed by luciferase reporter assay. ISL1 activated the promoter of c-Myc (E) or CyclinD1 (F) in a dose-dependent manner. The data represent 3 independent experiments, each performed in triplicate. Each bar represents mean ± SD (**p<0.01, *p<0.05, vs. the control).

Several cell cycle regulators, including CyclinD1, c-Myc, and CDK4 have been shown to control pancreatic islet cell proliferation *in vivo* [20], [21]. To identify the mechanism of ISL1-stimulated pancreatic islet cell proliferation, we tested whether the expression of these established cell cycle regulators was controlled by ISL1. HIT-T15 cells stably expressing ISL1 were subjected to real-time RT-PCR analysis for the expression of c-Myc, CyclinD1, CyclinA and p53. The results indicated that the overexpression of ISL1 did not significantly affect the expression levels of CyclinA and p53, but led to a three-fold increase in CyclinD1 expression and approximately two folds increase in c-Myc expression, relative to the control (Fig. 5A, 5B). Consistently, the knockdown of ISL1 did not alter the expression level of CyclinA, but was associated with the decrease in CyclinD1 expression level by 60% and in c-Myc expression level by 50% as compared with that in the cells transfected with non-silencer siRNA (Fig. 5C, 5D). Interestingly, the knockdown of ISL1 significantly enhanced the expression of p53 (Fig. 5C). To investigate the biological significance of ISL1-promoted cell proliferation, we then assessed the trans-activation activity of ISL1 on c-Myc or CyclinD1 promoters. In these experiments, HIT-T15 cells with ISL1 overexpression were transfected with a luciferase reporter construct: firefly luciferase gene inserted with c-Myc or CyclinD1 promoter. The results showed that the activation of c-Myc or CyclinD1 promoter exhibited a dose dependent manner within 1 µg of ISL1 with a constant amount of 0.2 µg c-Myc (Fig. 5E) or 1 µg of ISL1 with a constant amount of 0.2 µg CyclinD1 (Fig. 5F). These results indicate that ISL1 is able to activate the c-Myc and CyclinD1 promoters efficiently. Taken together, ISL1 promotes pancreatic islet cells proliferation possibly through the activation of the c-Myc and CyclinD1 promoters and thus increasing the expression of c-Myc and CyclinD1.

### ISL1 activated CyclinD1 or c-Myc transcription by binding to an evolutionarily conserved site

**Figure 6:**
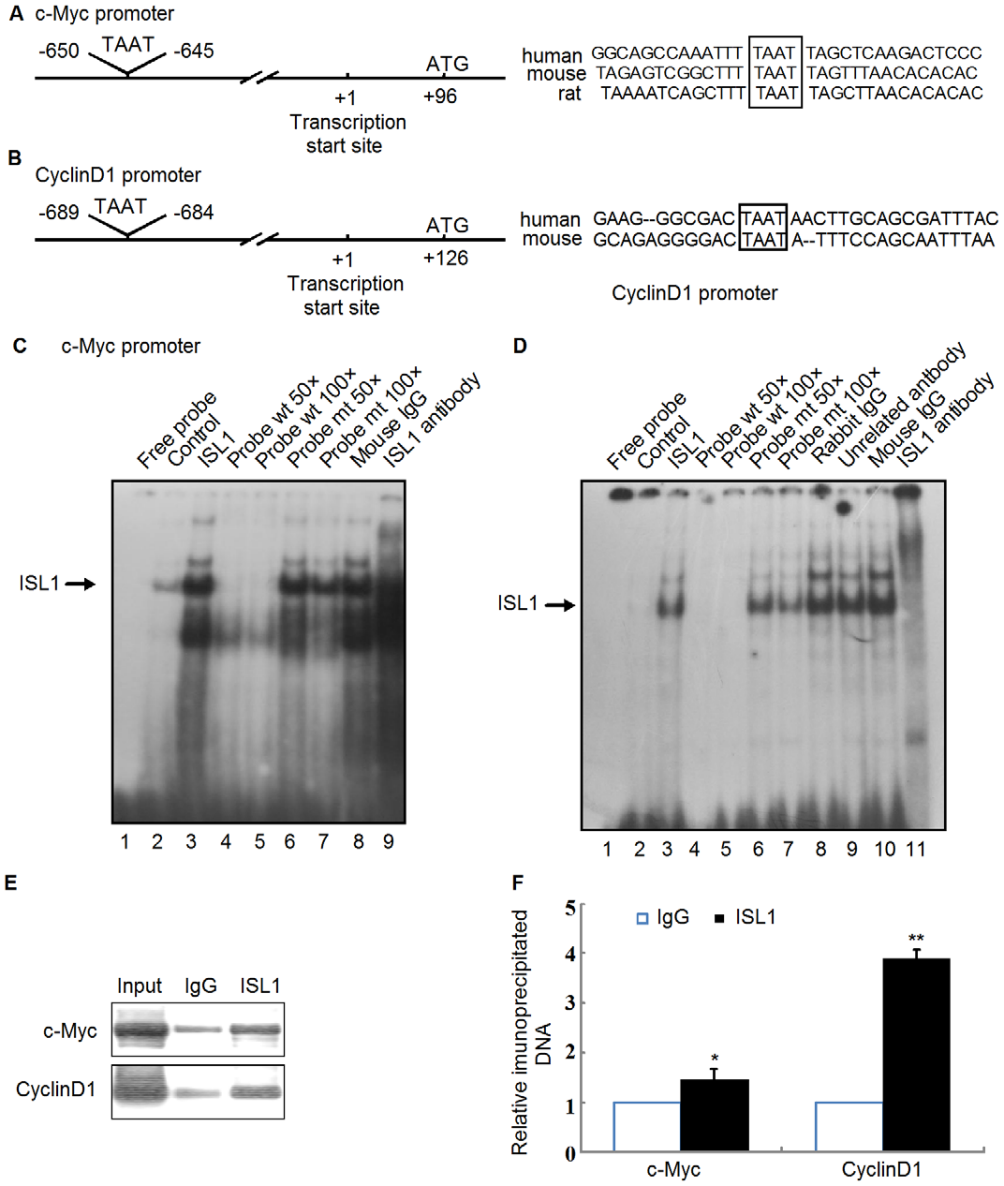
ISL1 band on the c-Myc or Cyclin D1 promoter directly. Consensus binding site (TAAT, box) for ISL1 on the c-Myc (A) or CyclinD1 (B) promoter was analyzed by Matinspector software. (C and D) Nuclear extracts were subjected to EMSA for the ISL1 proteins binding ability to the ^32^P-labeled oligonucleotides containing the consensus sequence of the c-Myc or CyclinD1 promoter. (C) Lane 1, free probe. Lane 2, nuclear extracts from HIT-T15 cell without transfection. Lanes 3–9, nuclear extracts from HIT-T15 cells transfected with ISL1 expression construct. Lane 3 shows the direct binding of ISL1. Lanes 4 and 5 show wild-type unlabeled oligonucleotide competition. Lanes 6 and 7 show mutant unlabeled probe competition. Lane 8, the mouse normal IgG was used as a negative control. Lane 9, 1 µg anti-ISL1 antibody (H00003670-M05, Abnova) was added. (D) Lanes 1–7, same as C, Lane 8, rabbit normal IgG was used as a negative control. Lane 9, 1 µg unrelated rabbit anti-GATA4 was added. Lane 10, mouse normal IgG was used as a negative control. Lane 11, 1 µg mouse anti-ISL1 polyclonal antibody was added. (E and F): ISL1 recruited on the c-Myc or Cyclin D1 promoter was analyzed by ChIP assay. Soluble chromatin was prepared from HIT-T15 cells stably transfected with 2 µg of pcDNA3.1-ISL1 plasmid followed by immunoprecipitation with antibodies against ISL1. The DNA extractions were amplified using the primers that cover the ISL1 binding sites on the c-Myc or Cyclin D1 promoter by PCR (E) or real-time PCR (F) with normal IgG as a control. The data represent 3 independent experiments, each performed in triplicate. Each bar represents mean ± SD (**p<0.01, *p<0.05, vs. the control).

We have shown that ISL1 could act as a transcriptional activator of c-Myc or CyclinD1. It is unknown whether ISL1 could directly control c-Myc or CyclinD1 transcription. Bioinformatic analysis with MatInspector software revealed a conserved ISL1 binding sequence (TAAT) 645 bp upstream of the ATG translation start site on the c-Myc promoter (Fig. 6A) and 684 bp upstream of the ATG translation start site on the CyclinD1 promoter (Fig. 6B). A region covering the ISL1 binding site located between −645 and −650 bp in mouse c-Myc promoter (Fig. 6A Left) or at −684 to −689 bp in mouse CyclinD1 promoter (Fig. 6B Left) was amplified using PCR and was used as a probe for subsequent electrophoretic mobility shift assays (EMSA). The results showed that a specific complex was formed with c-Myc (Fig. 6C, lane 3) or CyclinD1 (Fig. 6D, lane 3) probe. The complex specificity was confirmed by performing incubation with a 100-fold molar excess of unlabeled oligonucleotide prior to the addition of the labeled probes (Fig. 6C, lanes 4–5 and Fig. 6D, lanes 4–5). The mutation of ISL1 binding site on c-Myc or CyclinD1 probe failed to compete with the complex formation (Fig. 6C, lanes 6–7 and Fig. 6D, lanes 6–7). The addition of normal rabbit IgG, mouse IgG and the unrelated antibody did not affect the protein-DNA complex formation (Fig. 6C, lane 8 and Fig. 6D, lanes 8–10), while the complex bands were dramatically attenuated in the presence of anti-ISL1 antibody, further proving the specificity of the protein-DNA complex (Fig. 6C, lane 9 and Fig. 6D, lane 11).

Promoter chromatin immunoprecipitation (ChIP) assay was performed in HIT-T15 cells to determine if ISL1 could occupy the c-Myc promoter or CyclinD1 promoter region *in vivo*. Results show that the ISL1 antibody specifically immunoprecipitated with the DNA fragments containing the c-Myc promoter or CyclinD1 promoter in ISL1 overexpressing cells. As shown in Fig. 6E, the overexpression of ISL1 significantly accentuated the binding of ISL1 on the c-Myc or CyclinD1 promoter compared to IgG, suggesting that ISL1 could bind on the c-Myc or CyclinD1 promoter *in vivo*. As shown in Fig. 6F, ISL1 was recruited to the Cyclin D1 promoter four folds as compared with lgG, whereas the binding level on the c-Myc promoter was less than 1.5 folds relative to IgG. Collectively, these *in vitro* and endogenous binding data indicate that ISL1 is a direct regulator of c-Myc and CyclinD1 transcription in pancreatic β cells.

## Discussion

ISL1 is a LIM-homeodomain transcription factor and plays important roles in the early development of heart, motor neurons and pancreas at embryonic stage. The expression of ISL1 is down-regulated in heart cells after birth but remains at a high level in normal adult islet cells, indicating that ISL1 may have some functions in terminally differentiated islets. Previous studies provided evidences that ISL1 is required for proliferation of human cardiac progenitor cells and motor neurons in injured zebrafish [13], [14]. Furthermore, Du A et al. [16] demonstrated the requirement of ISL1 in the maturation, proliferation, and survival of hormone-producing islet cells after the secondary transition and in postnatal lives, indicating a crucial role of ISL1 in regulating endocrine cell growth and survival in young animals [16]. However, whether ISL1 can prevent islet cells from apoptosis or promote mature islet cell proliferation are still questions to be answered.

Our results show that ISL1 knockdown in HIT-T15 cells could increase the level of apoptosis even in normal cell culture condition. However, the level of apoptosis was dramatically increased in response to the glucose oxidase stimulation, suggesting that ISL1 may play roles in anti-apoptosis under the oxidative stress. Moreover, our study demonstrated that ISL1 could promote mature pancreatic islet cell proliferation and identified several downstream targets for ISL1 in the islets. It was reported that ISL1 could directly bind to MafA in the early stage of pancreas development [16], but its direct downstream targets in adult pancreatic islets were uncharacterized. As the adult islet cell proliferation is a complex process and requires a dynamic change to meet the requirement of pancreatic function, the regulation of the islet cell proliferation must result from the orchestration of many transcriptional factors and growth factors. CyclinD1, CyclinD2, CDK4, and c-Myc, amongst others, have been reported to control the islet cell proliferation during pancreas development [20], [21]. We investigated whether the expression of these established cell cycle regulators are controlled by ISL1. As a transcription factor, ISL1 exerts its function by binding to a consensus sequence TAAT on the promoter of target genes. Our promoter chromatin immunoprecipitation (ChIP) assay results revealed that ISL1 could regulate the transcription of c-Myc and CyclinD1 by directly binding on the c-Myc or CyclinD1 promoter *in vivo*. Furthermore, the luciferase assays demonstrated that the overexpression of ISL1 was positively correlated with the promoter activity of c-Myc or CyclinD1 promoter, while knockdown of ISL1 inhibited their activity.

Wild-type p53 acts as a potent regulator of the cell cycle, especially coordinating transcriptional responses to pathological stress [22]. In response to DNA damage, p53 accumulates in cell nuclei causing cell arrest at the G_1_ phase and inducing apoptosis [23], [24]. We found that the overexpression of ISL1 did not affect the expression level of p53, but the knockdown of ISL1 significantly enhanced the expression of p53 and increased the apoptosis. Whether p53 is involved in the anti-apoptosis role of ISL1 in the islet cells requires further investigation. It should be mentioned that we have observed that ISL1 overexpression also slightly increased the level of apoptosis of HIT-T15 cells (data not shown). This phenomenon is possibly due to the high level of endogenous ISL1 expression in HIT-T15 cells and the overexpression of ISL1 may disrupt the orchestration of ISL1 regulation. We have noticed that EdU incorporation rate in ISL1 expressing adult islet cells is about 2.5 folds than that in the control (Fig. 3C, 3E). However, the differences are not so obvious in FCM analysis. EdU incorporation and FCM analysis are two different methods reflecting cell proliferation. EdU incorporation detected the newly synthesized random DNA regardless of cell cycle phase transition. The data are expressed as folds of positive islet cells relative to negative cells. While FCM detected the cell proliferation, represented by S/G_2_M phases transition in one cell cycle. Additionally, as ISL1 target genes, we also noted CyclinD1 was regulated more significantly than c-Myc by ISL1. This implies that CyclinD1 might be a more potent downstream factor to mediate the ISL1 proliferation effects in pancreatic islet cells.

In conclusion, ISL1 can promote mature pancreatic islet cells proliferation and attenuate cell apoptosis against oxidative stress. c-Myc and CyclinD1 are identified as novel downstream targets of ISL1 and are involved in ISL1 regulation on the proliferation of adult islet cells. Our findings extend the knowledge about the crucial role of ISL1 in maintaining mature islet cells homeostasis. Our results also provide insights into the new regulation relationship between ISL1 and other growth factors.

## Materials and Methods

### Ethics Statement

The animal experiments were performed in accordance with the ethical principles and guidelines for scientific experiments on animals of the Swiss Academy of Medical Sciences (1995). All protocols were approved by the Animal Care and Use Committee of Peking University (LA 2010-066). The animals in the postprandial state were anesthetized with 5 mg/100 g body weight of sodium pentobarbital and pancreatic tissue was removed.

### Plasmid constructs and recombinant lentivirus

The plasmid construct pcDNA3.1-ISL1 was previously described [25] and was used as stable transfection. The pLL3.7-ISL1-siRNA plasmid was commercially constructed by the GeneChem Company (Shanghai, China) and was used for stable transfection. The luciferase reporter constructs c-Myc-luc and CyclinD1-luc were generous gifts from Prof. Yongfeng Shang (Department of Biochemistry and Molecular Biology, Peking University Health Science Center).

The sequences of ISL1-siRNA are: sense, GAGACAUGGUGGUUUAtt; antisense: UUUCUCCUUGCACCUCtt. The sequences of non-silencer siRNA are: sense: UUCUCCGAACGUGUCACGUtt; antisense: ACGUGACACGUUCGGAGAAtt. Recombinant lentiviruses with ISL1 cDNA or ISL1-siRNA were obtained from the Genechem Company (Shanghai, China). Lentivirus infection was carried out following the manufacturer's instruction with multiplicity of infection as 10 and rat adult islet cells were used as target cells. The infection efficiency was detected by flow cytometry analysis.

### Cell cultures, stable cell lines and adult islets isolation

Monolayer cultures of hamster pancreatic islet β cell line HIT-T15 (ATCC number: CRL-1777) were maintained in RPMI 1640 (GIBCO BRL) complete medium, which contained 2 mM/L glutamine, 100 U/ml penicillin, 100 U/ml streptomycin, and 10% fetal bovine serum. The medium was changed every 1–2 days.

To establish stable cell lines, 2×10^5^ HIT-T15 cells were plated into a 60 mm culture dish. When approximately 50% confluence was reached, HIT-T15 cells were transfected with 2 µg pcDNA3.1-ISL1 plasmid, or a control pcDNA3.1 plasmid, respectively, with Lipofectamine2000. G418 selection (1000 µg/ml) was performed and single colonies were picked up at about 21 days. The cells transfected with pLL3.7-ISL1-siRNA or pLL3.7-non-silencer were selected using puromycin resistant screening (1 µg/ml). Identification of stable cell lines were performed using RT-PCR and Western blotting for quantifying the expression levels of ISL1.

Pancreatic islets were isolated from 100–120 g male Sprague-Dawley rats as previously described [26]. Briefly, donor pancreases were perfused *in situ* with collagenase V (3 mg/ml, Sigma-Aldrich, St. Louis, MO, USA), and the pancreatic tissues were harvested after the perfusion and were further incubated at 37°C with gentle vortex (375 rpm) for 30 min. Islets were released from the pancreas and were handpicked. The isolated islets were washed with Hank's solution twice at 4°C, counted and cultured in RPMI complete medium.

### Cell proliferation assays and clone formation assay

The pancreatic cell proliferation was measured by performing WST-8 assay and 5-ethynyl-20-deoxyuridine (EdU) incorporation assay, using a CCK-8 cell proliferation kit (Dojindo Laboratories, Kumamoto, Japan) and EdU assay kit (Ribobio, Guangzhou China), respectively, according to the manufacturers' instructions. For CCK-8 assay, cells were seeded into a 96-well plate at 1×10^3^cells per well with 100 µl complete medium and cultured at 37°C, 10 µl CCK-8 solution was added to each well after 12 h, 24 h, 48 h, and 72 h, respectively. Plates were incubated at 37°C for 2 h, and then the absorbance at 450 nm was measured with Microplate Reader (Bio-Rad, La Jolla, CA, USA). All experiments were done in triplicate and three independent repeating experiments were performed.

For EdU incorporation assay, cells were cultured in triplicate in 96-well plates at a density of 1×10^3^ HIT-T15 cells or 50 rat islets mass per well for 48 h at 37°C, and then 50 µM of EdU was added to each well and cells were cultured for additional 4 h at 37°C. The cells were fixed with 4% formaldehyde for 15 min at room temperature and treated with 0.5% Triton X-100 for 20 min at room temperature for permeabilization. After washing with PBS three times, 100 µl of 1× Apollo® reaction cocktail was added to each well and the cells were incubated for 30 min at room temperature. Then the cells were stained with 100 µl of Hoechst33342 for 30 min and visualized under a fluorescent microscope (Olympus Corporation, Tokyo, Japan). The EdU positive cells (red cells) were counted using Image-Pro Plus (IPP) 6.0 software (Media Cybernetics, Bethesda, MD, USA). The EdU incorporation rate was expressed as the ratio of EdU positive cells to total Hoechst33342 positive cells (blue cells). All experiments were done in triplicate and three independent repeating experiments were performed.

A total of 1×10^5^ ISL1 or ISL1-siRNA stable transfection cells were plated onto a 60-mm culture dish. Cells were maintained in culture medium supplemented with 1 mg/ml G418 or 1 µg/ml puromycin for 21 days and stained with crystal violet for colony counting. The colony diameter greater than 4.5 mm was counted using IPP 6.0 software. All experiments were done in triplicate and three independent repeating experiments were performed.

### Flow cytometry analysis for cell cycle, apoptosis and ROS generation

Cell cycle analysis was performed by flow cytometry. Briefly, cultured cells were trypsinized into single cell suspensions and fixed with 70% ethanol for 30 min on ice. RNA was degraded by incubation with 20 mg/ml RNase (Sigma-Aldrich, St. Louis, MO, USA) for 1 h at 37°C. DNA was labeled with 20 mg/ml propidium iodide (PI, Sigma-Aldrich) and DNA content was assessed by FACS Calibur flow cytometry (Becton Dickinson, Franklin Lakes, NJ, USA) equipped with the ModiFit LT v2.0 software. For apoptosis analysis, cultured cells were harvested by trypsinization and washed with PBS. 1×10^6^ cells from each sample were processed for Annexin V FITC/PI apoptosis detection (Becton Dickinson) according to the manufacturer's instructions.

Reactive oxygen species (ROS) were detected using reactive oxygen species assay kit C1300 (Applygen, Beijing, China). Cells were incubated with variable concentrations of glucose oxidase (0 to 100 mU/mL) in fresh serum-free RPMI1640 for 4 h, before the absorbance at 530 nm was assessed by FACS Calibur flow cytometry. Data are presented as the percentage of ROS positive cells amongst total cells. All experiments were done in triplicate and three independent repeating experiments were performed.

### RT-PCR and Real-time RT-PCR

Cells were seeded into a 6-well plate at 1×10^5^ cells per well and harvested after 48 h culture. Total RNA extraction was performed using Trizol Reagent (Invitrogen, Carlsbad, CA, USA) based on the manufacturer's instructions. Amplifications were performed in the ABI 7300 Real-Time RT-PCR System (Carlsbad, CA, USA) with different primers (Table 1). All annealing temperatures were 60°C. Transcription levels were normalized to 18S rRNA levels. Each value presents the average of at least 3 independent experiments.

**Table 1:** Primers used in RT-PCR and real-time RT-PCR.

Primers	Primer sequences (5′–3′)	Product size (bp)
18SRNA	F: GTAACCCGTTGAACCCCATT	151
	R: CCATCCAATCGGTAGTAGCG	
ISL1	F: CTGCTTTTCAGCAACTGGTCA	123
	R: TAGGACTGGCTACCATGCTGT	
c-Myc	F: GCCACGTCTCCACACATCAG	141
	R: TCTTGGCAGCAGGATAGTCCTT	
CyclinD1	F: GCGTACCCTGACACCCCTCTC	183
	R: CTCCTCTTCGCCTGATCC	
CyclinA	F: GCCTTCACCATTCATGTGGAT	118
	R: TTGCTGCGGGTAAAGAGACAG	
p53	F: TTCCACCTGGGCTTCCTG	144
	R: GGATAGGTCGGCGGTTCAT	
Insulin	F: AGGACCCACAAGTGGAACAACT	140
	R: CAACGCCAAGGTCTGAAGGT	

### Western blotting

Western blotting was performed as previously described [27]. Cells were seeded into a 6-well plate at 1×10^5^cells per well and were harvested after 48 h culture. Total protein was prepared and subjected to 12% SDS polyacrylamide gel electrophoresis and subsequently transferred onto nitrocellulose membranes. Antibodies, including ISL1 (H00003670-M05, Abnova, Taipei, China), CyclinD1 (ab61758, Abcam, Hong Kong, China), c-Myc (sc-764, Santa Cruz, CA, U.S.A.) and horseradish peroxidase-conjugated secondary antibody from Santa Cruz were used.

### Luciferase assays

HIT-T15 cells were seeded at a density of 10×10^4^ per well in a 24-well plate, constructs of c-Myc-luc, CyclinD1-luc, or ISL1 were transfected using Lipofectamine™2000 (Invitrogen, Carlsbad, CA, USA) according to the manufacturer's instructions when cells reached 60% confluence. The total amount of DNA was kept constant using pcDNA3.1/β-gal plasmid. Luciferase activity was measured and normalized to Renilla luciferase activity. All experiments were done in triplicates and three independent repeating experiments were performed.

### Electrophoretic mobility-shift assay (EMSA)

Nuclear extracts were prepared from HIT-T15 cells. Oligonucleotides used in EMSA assay were labeled with [γ-^32^P] ATP as described previously [27]. The sequences of the sense strand of these oligonucleotides are as follows (ISL1 binding motif is under-lined and mutant bases are in lower cases):

Wild-type c-Myc probe: 5′-ATTAGAGTCGGCTTTTAATTAGTTAACACACAC-3′.

Mutant c-Myc probe: 5′-ATTAGAGTCGGCTTTgccgTAGTTAACACACAC-3′.

Wild-type CyclinD1 probe: 5′-AATTTAATTTCTTTTTTAATTAAAAAAAATGAGT C-3′. Mutant CyclinD1 probe: 5′-AATTTAATTTCTTTTTgccgTAAAAAAAATGA GTC- 3′. The binding reactions were performed at 4°C (20 µl final volume) with 10 µg of nuclear protein and 0.25 ng (20 kcpm) of radiolabeled, double-stranded oligonucleotide in a binding buffer. Antibody analyses were performed by pre-incubation of nuclear extract protein with specific ISL1 antibody (Abnova) for 20 min at 4°C prior to addition of the radiolabeled probe. Protein-DNA complexes were resolved on a 6% native polyacrylamide gel and visualized by autoradiography.

### ChIP assay

HIT-T15 cells were stably transfected with 2 µg of pcDNA3.1-ISL1 plasmid and cells were harvested after 48 h culture. ChIP experiments were performed according to the method described previously [17]. After crosslink reversal, precipitated DNA was analyzed by PCR for fragments of the c-Myc and CyclinD1 promoters with different primers. The annealing temperatures were 60°C. The input DNA and immunoprecipitated DNA were calculated by real-time PCR using SYBR® Green Real-time PCR Master Mix (TOYOBO, Japan). The data obtained were normalized to the corresponding DNA precipitated by IgG. c-Myc: forward (F), 5′-AATGCACAGCGTAGTATTCAGGA-3′; reverse (R), 5′ GGA GTG AAT TGC CAA CCC AGA 3′ (270 bp fragment). CyclinD1: forward (F), 5′- AGCTTCGGTGTCTGGTTC- 3′; reverse (R), 5′- ATTCCAGCAACGCTCAA GATG- 3′ (283 bp fragment).

### Type 1 and type 2 diabetes animal models

STZ-induced diabetic mice (male C57BL/6 mice at the age of 8 to 10 weeks, obtained from Department of Laboratory Animal Science, Peking University, China), were established as described previously [17]. The mice with stable hyperglycemia (blood glucose level >20 mmol/L) were used as the type 1 diabetes animal model. Akita, db/db and db/w mice were provided by Prof. Youfei Guan (Peking University, China). The Akita mice at 12 weeks of age were used as an autosomal dominant mutation Mody animal model and showed hyperglycemia with notable pancreatic β-cell dysfunction. Db/db mice at 14–16 weeks of age were used as the type 2 diabetes animal model, which were homozygous for the db gene and, thus, exhibited an obese, diabetic phenotype. Db/w mice that were heterozygous for the db gene exhibited a nondiabetic, normal phenotype were used as controls to db/db mice. The type 1 and type 2 diabetes mice in the postprandial state were anesthetized with 5 mg/100 g body weight of sodium pentobarbital and pancreatic tissue was removed. RNA was prepared from these tissues and used for real-time RT-PCR assay.

### Statistical analysis

The data are expressed as mean ± standard deviation (S.D.). Comparisons between groups were analyzed using Student's *t*-test or ANOVA, and the Student-Newman-Kleuss method was used to estimate the level of significance. Differences were considered to be statistically significant at *p*<0.05.

## Supporting Information

Figure S1 **Glucose oxidase stimulated ROS production.** Level of ROS production was measured by flow cytometry analysis. HIT-T15 cells were treated with glucose oxidase (GO) at various concentrations (0–100 mU/mL) for 4 h. ROS production exhibited a dose dependent manner with GO concentration.
(TIF)

Figure S2 **Glucose oxidase increased the number of apoptotic cells.** Level of apoptosis rate was measured by flow cytometry analysis. HIT-T15 were treated with GO at various concentrations (0–100 mU/mL) for 4 h. the numbers of apoptotic cells increased in a dose dependent manner with GO concentration.
(TIF)
