# Supplementary figures and images for: ISL1 Promotes Pancreatic Islet Cell Proliferation
Source: PLoS One. 2011 Aug 4;6(8):e22387. doi: 10.1371/journal.pone.0022387 (PMC3150357; doi:10.1371/journal.pone.0022387)

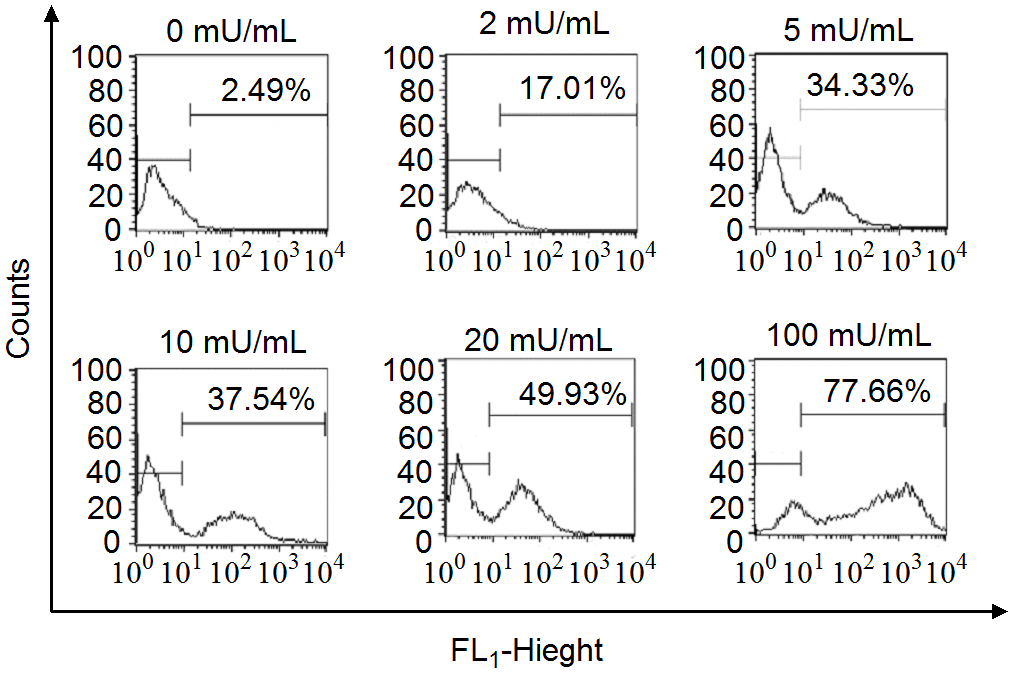

Supplement: Figure S1 — Glucose oxidase stimulated ROS production. Level of ROS production was measured by flow cytometry analysis. HIT-T15 cells were treated with glucose oxidase (GO) at various concentrations (0–100 mU/mL) for 4 h. ROS production exhibited a dose dependent manner with GO concentration. (TIF) [file pone.0022387.s001.tif]

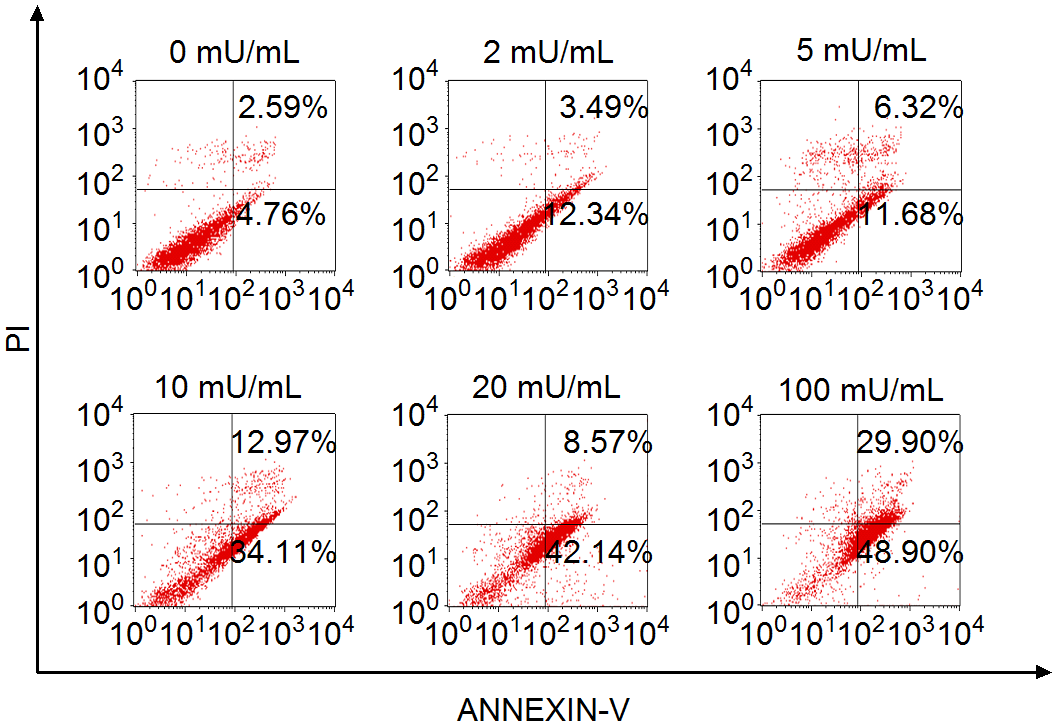

Supplement: Figure S2 — Glucose oxidase increased the number of apoptotic cells. Level of apoptosis rate was measured by flow cytometry analysis. HIT-T15 were treated with GO at various concentrations (0–100 mU/mL) for 4 h. the numbers of apoptotic cells increased in a dose dependent manner with GO concentration. (TIF) [file pone.0022387.s002.tif]
